# Injuries Related to Waterskiing Between 2012 and 2022: A National Database Study

**DOI:** 10.7759/cureus.65522

**Published:** 2024-07-27

**Authors:** Sean M Muir, Tyler Rizzieri, Andrew Brown, Cole Graham, Laken Fulmer, Ryan Scagliarini, Kristine Lombardozzi

**Affiliations:** 1 Spine Surgery, Steadman Philippon Research Insitute, Vail, USA; 2 Residency, OhioHealth Riverside Methodist Hospital, Spartanburg, USA; 3 Medicine, Edward Via College of Osteopathic Medicine, Spartanburg, USA; 4 Surgery, Spartanburg Regional Medical Center, Spartanburg, USA; 5 Surgery, VCOM-Carolinas, Spartanburg, USA

**Keywords:** health surveillance, head injuries, athletic injuries, epidemiology, water skiing

## Abstract

We conducted an epidemiological study by using the National Electronic Injury Surveillance System (NEISS) database to assess and quantify waterskiing injuries between 2012 and 2022. A total of 898 injuries were reported, primarily among Caucasian males during competition. Head (177, 20%), face (93, 10%), and knee (70, 8%) were the most prevalent types of injuries. Common physical examination findings included muscle strains, ligament sprains, lacerations, and fractures. Although rare, there were reported cases of dental injury, hemorrhage, crushing, foreign body, and amputation as well. These findings suggest that protective equipment, particularly headgear, is necessary to reduce the incidence of waterskiing injuries.

## Introduction

Waterskiing was introduced in the early 1920s in Lake Pepin, Minnesota, with a simple ski, tow rope, and boat [1-3]. The American Water Ski Association (AWSA) was founded in 1939, and the sport gained wide popularity post-World War II [3,4]. The International Waterski & Wakeboard Federation (IWWF) was established in 1946 and is recognized by the International Olympic Committee (IOC) [5]. Technological advancements in both boats and skis have contributed to the sport’s rapid growth and the development of more than 30 waterskiing disciplines [4,6]. Although mostly a recreational sport, waterskiing has been a part of the Olympic Games since 1972 as well as the Pan American Games since 1995 [7]. The sport has nationally recognized teams, such as the USA Waterski and Wake Sports Team, that compete globally [4].

Popular disciplines within waterskiing include slalom skiing, trick skiing, and jump skiing [4]. Slalom skiing is a timed sport that involves navigating through a course of buoys set at predetermined distances [4,8]. It requires extreme balance and focus to maintain speed and control [8]. The tow rope is shortened, and the speed of the boat is increased after each buoy with the objective of completing it around as many buoys as possible [4,8]. Participants score points based on speed, rope distance, and buoys passed [4]. Trick skiing focuses less on speed and more on the athlete’s creativity, strength, and flexibility [4,8]. Athletes have a set time to perform a unique variety of tricks, spins, and flips similar to half-pipe snow skiing [4]. They are given points based on difficulty, creativity, and completion of the trick [4]. Lastly, water jump skiing involves using slender skis and boat momentum to launch off a jump. The objective is to get the maximum flight distance. Forward or backward rotation, known as “cutting”, helps to lift the skier and increase flight distance [4]. Skiers can reach speeds up to 70 mph before launching off the ramp [4].

Similar to many sports or recreational activities, waterskiing is associated with various injuries. In this study, we examined the National Electronic Injury Surveillance System (NEISS) to quantify and categorize waterski-related injuries that resulted in emergency room visits between 2012 and 2022. Based on our findings, we recommend adjustments in the types of personal protective equipment currently used to reduce or prevent waterskiing injuries.

## Materials and methods

NEISS, managed by the United States Consumer Product Safety Commission (CPSC), was used to determine the number and type of waterskiing injuries requiring emergency room visits between 2012 and 2022 [9]. NEISS is a statistically validated injury surveillance system that comprehensively collects and reports national emergency department visits linked to consumer products or toxicity. The database is user-friendly and allows individuals to tailor queries based on various parameters such as year of injury, gender, age, diagnosis, location, affected body part, mechanism of injury, and the product involved.

NEISS gathers data from over 100 hospitals with a minimum of six beds and 24-hour emergency department services. These hospitals submit daily injury reports, representing a stratified sample based on emergency department size and geographic location. The NEISS database comprises a sample of hospitals but does not provide the total number of injuries. Using a coefficient of variation to estimate the confidence interval, approximately 47,929 patients (95% CI: 34,266-61,592) in the United States sought emergency treatment for waterskiing injuries between 2012 and 2022.

Ninety-three standardized NEISS codes were used to classify injuries anatomically. These codes were translated into categories including internal organ injuries, shoulder/upper arm, elbow/forearm, hand/wrist, head/neck, chest, pelvis, hip/upper lower extremity, knee/leg, and foot/ankle. Injuries affecting multiple anatomical locations were identified and classified into lower extremity, head/neck, trunk, upper extremity, and general bodily injuries. The axial body consisted of the chest, belly, pelvis, and spine, while the appendicular body comprised the upper and lower extremities.

Only waterskiing cases where the injured individuals sought treatment in emergency departments and were recorded in NEISS between January 1, 2012, and December 31, 2022, were analyzed. Injuries across all age groups, genders, and geographical locations within the United States were considered. Any type of injury diagnosis related to waterskiing, such as sprains, strains, fractures, and lacerations, was included. Injuries associated with other water sports such as wakeboarding, tubing, or jet skiing were excluded. Injuries treated outside of emergency departments, such as in outpatient clinics or through self-treatment, were also excluded. Cases with insufficient data to accurately determine the nature or context of the injury, such as those with missing key demographic information or unclear injury mechanisms, were omitted. Duplicate entries of the same injury incident were removed to avoid redundancy.

## Results

**Figure 1:**
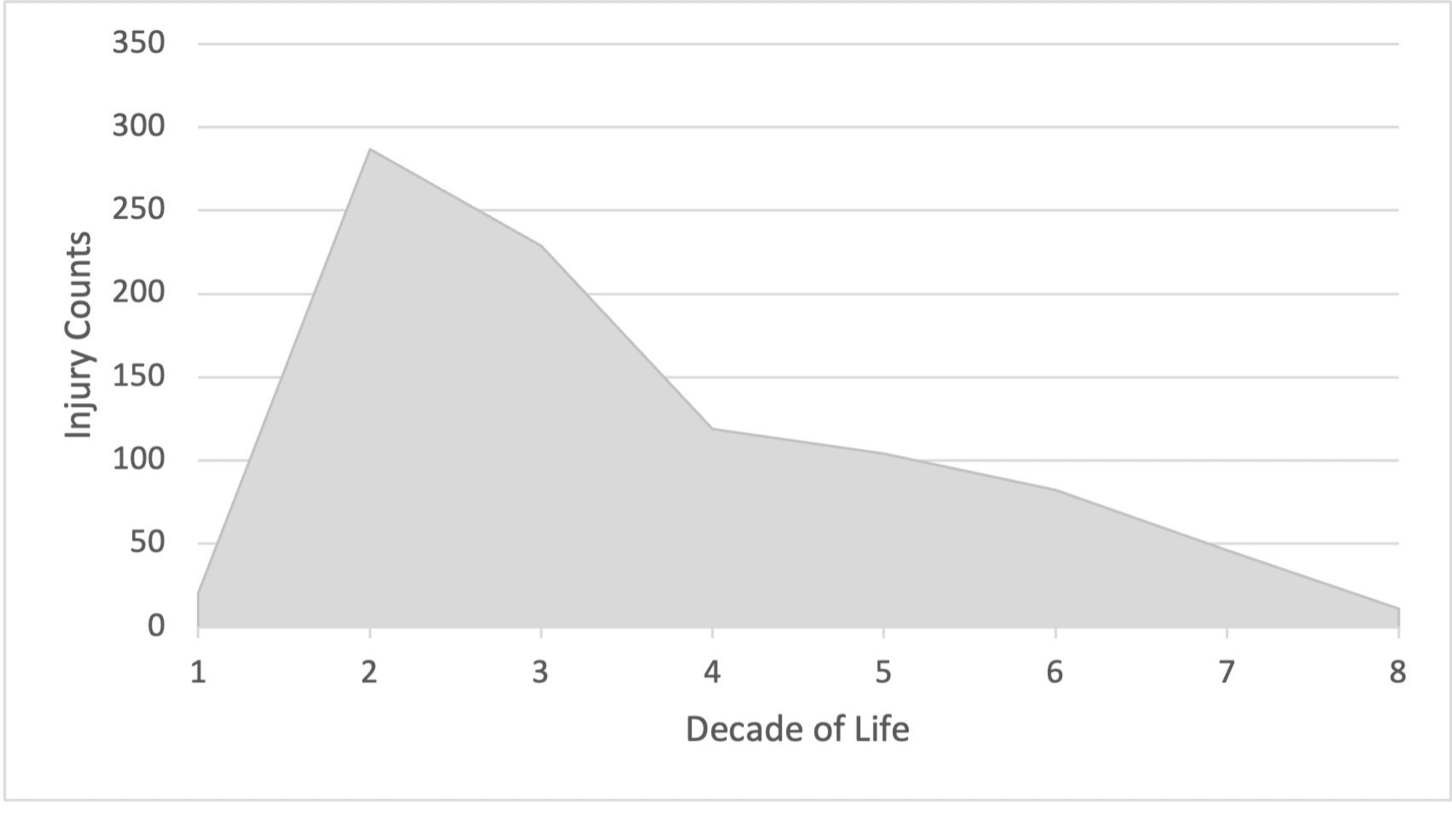
Injuries by Decade of Life

**Figure 2:**
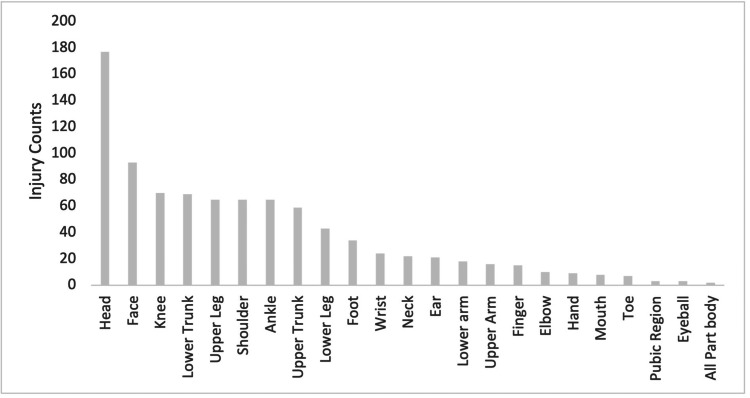
Injury Count by Body Part

**Figure 3:**
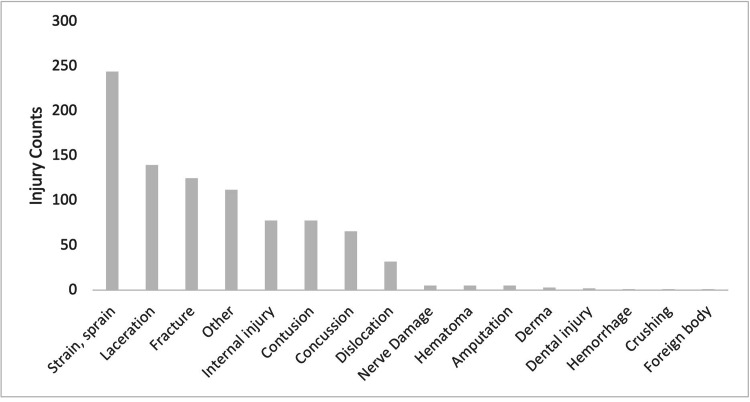
Injury Count by Diagnosis

A total of 898 reported waterskiing-related injuries resulted in emergency room visits between 2012 and 2022 (Table 1). Of them, 274 were females and 624 were males. Individuals in their second decade of life (10-19 years of age), accounted for 32% of the injuries (Figure 1). The majority of injuries that occurred during sporting events involved the head (177 injuries), face (93 injuries), and knee (70 injuries; Table 2, Figure 2). Sprains and strains (244), lacerations (140), and fractures (125) were the most commonly diagnosed injuries overall (Table 3, Figure 3). The majority of individuals were Caucasian (563), and 310 individuals did not report their ethnicity. Ten individuals were classified as 'others', eight as Black, four as Asian, two as American Indian, and one as Native Hawaiian.

**Table 1:** Study Demographics

Category	Subcategory	Counts
Sex	Male	274 (31%)
	Female	624 (69%)
	Total	898
Race	White	563 (63%)
	Black	8 (<1%)
	Asian	4 (<1%)
	American Indian	2 (<1%)
	Native Hawaiian	1 (<1%)
	Other	10 (1%)
	Not Specified	310 (35%)
Injuries by Age Decade	1st Decade (0-9 years)	20 (2%)
	2nd Decade (10-19 years)	287 (32%)
	3rd Decade (20-29 years)	229 (26%)
	4th Decade (30-39 years)	119 (13%)
	5th Decade (40-49 years)	104 (12%)
	6th Decade (50-59 years)	82 (9%)
	7th Decade (60-69 years)	46 (5%)
	8th Decade (70-79 years)	11 (1%)

**Table 2:** Injury Count Based on Body Part

Body Part	Count	Percentage
Head	177	19.80%
Face	93	10.40%
Knee	70	7.80%
Lower Trunk	69	7.70%
Upper Leg	65	7.30%
Shoulder	65	7.30%
Ankle	65	7.30%
Upper Trunk	59	6.60%
Lower Leg	43	4.80%
Foot	34	3.80%
Wrist	24	2.70%
Neck	22	2.50%
Ear	21	2.40%
Lower Arm	18	2.00%
Upper Arm	16	1.80%
Finger	15	1.70%
Elbow	10	1.10%
Hand	9	1.00%
Mouth	8	0.90%
Toe	7	0.80%
Pubic Region	3	0.30%
Eyeball	2	0.20%
All Body Parts	112	12.50%
Total	786	87.50%
Grand Total	898	100%

**Table 3:** Injury Count Based on Diagnosis

Diagnosis	Count	Percentage
Strain, sprain	244	27.20%
Laceration	140	15.60%
Fracture	125	13.90%
Other	112	12.50%
Internal injury	78	8.70%
Contusion	78	8.70%
Concussion	66	7.40%
Dislocation	32	3.60%
Total	875	97.40%
Nerve Damage	5	0.60%
Hematoma	5	0.60%
Amputation	5	0.60%
Derma	3	0.30%
Dental injury	2	0.20%
Hemorrhage	1	0.10%
Crushing	1	0.10%
Foreign body	1	0.10%
Total	23	2.60%
Grand Total	898	100%

## Discussion

The head was the most commonly injured body part, representing 177 (20%) of all emergency room visits. Of these, 65 (37%) were diagnosed as concussions, raising concern about subsequent head injury and long-term consequences. Further investigations into water sports head safety gear are warranted. Injuries to the face and knee were also common, with 93 and 70 reported cases, respectively. Further understanding of the mechanisms responsible for these injuries could guide targeted interventions to reduce their occurrence. For example, knee braces or guards are not currently considered a priority in waterski safety. Lower trunk, upper leg, shoulder, and ankle injuries were also prevalent, accounting for 69, 65, 65, and 65 cases, respectively. Injuries to the torso and thigh also suggest the need for lightweight ergonomically designed protective equipment for the trunk area.

Sprains and strains (244, 27%) comprised the largest diagnostic type and were likely related to sudden changes of direction or falls. Contact with waterskiing accessories (skis, bridle, rope) or collision with the water or floating and submerged obstacles may be the cause of the reported lacerations. The high frequency of broken bones (125, 14%) and rare diagnoses of amputation, dental injury, hemorrhage, crushing, and foreign body suggest inadequate preparation, high speeds, or carelessness. These diagnoses highlight the potential for severe and unique injuries associated with waterskiing accidents and underscore the importance of mandatory waterskiing safety courses and personal protective equipment especially for beginners.

Currently, athletes are always required to wear US Coast Guard-approved life jackets or personal flotation devices (PFDs) while competing in waterski sporting events [10]. The primary use of these devices pertains to flotation; however, recent efforts have focused on additional safety features to protect injury to the torso [10]. Combining PFDs with torso protection would be particularly beneficial for trick and jump skiing. Helmets, goggles, and hearing protection should be mandatory or strongly recommended. Additionally, the utility of a protective face mask to help prevent lacerations to the face should be investigated based on the number of face lacerations injuries (93, 17%) reported. Lastly, wetsuits, rash guards, gloves, and footwear are the other protective gear that we recommend.

**Figure 4:**
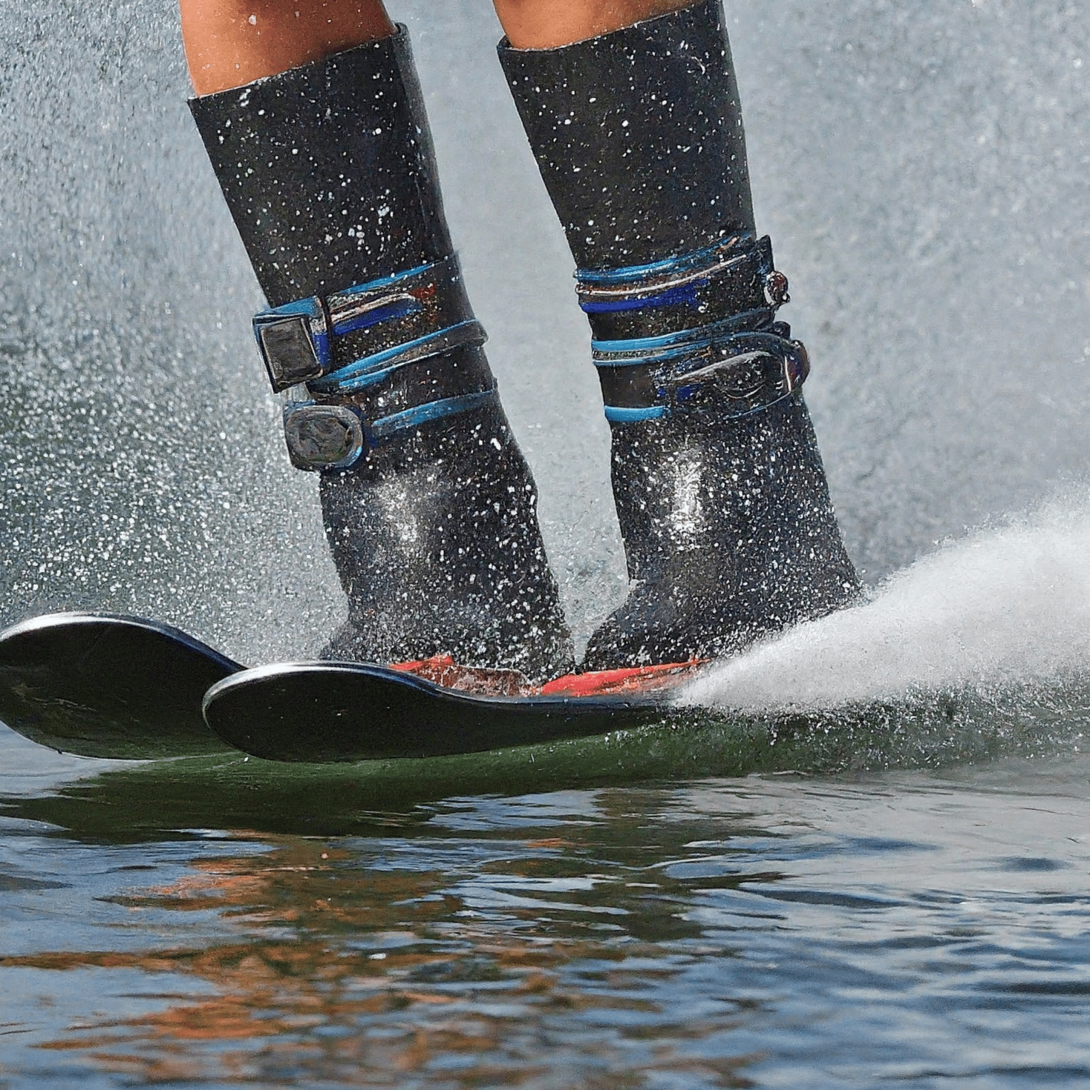
Closeup Image of Waterskiing A waterskier glides across the water on two skis, with the water rushing by beneath them. The skis are secured to the feet with bindings

**Figure 5:**
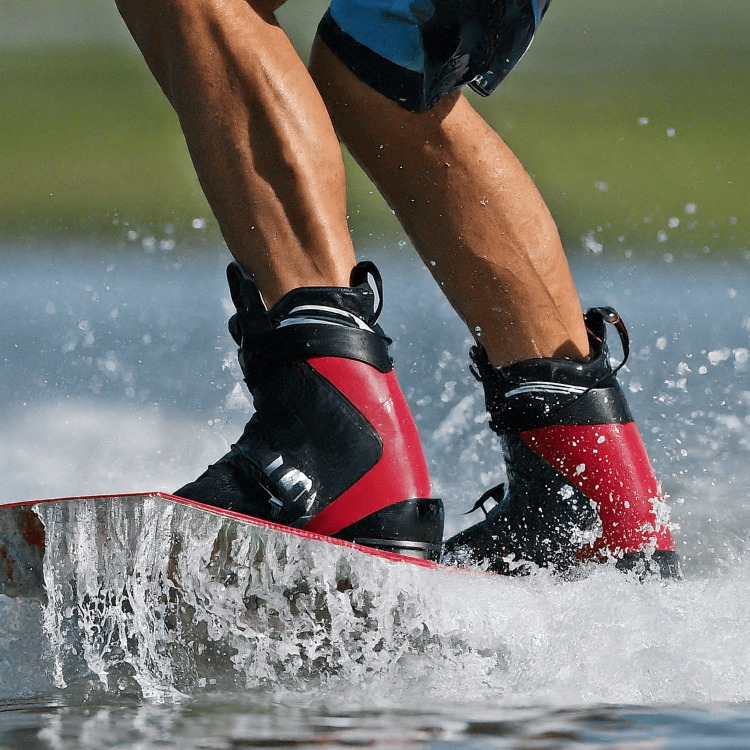
Closeup Image of Wakeboarding A wakeboarder skims across the water, riding a single board while the waves rush past. The feet are strapped into the board with bindings, keeping them securely in place as they carve through the water

Baker et al. (2010) collected data from 1,761 individuals involved in tubing, wakeboarding, and waterskiing injuries from the NEISS between 2000 and 2007 (Figures 4-5) [11]. Wakeboarding and tubing injuries frequently involved the head and neck, while waterskiing injuries more commonly involved the hip and lower extremities [11]. Interestingly, the study found that tubing-related injuries were more severe compared to waterskiing injuries [11]. A similar study by Hostetler et al. evaluated characteristics of waterskiing- and wakeboarding-related injuries between 2001 and 2003 [12]. They found that wakeboarder injuries were associated with a higher incidence of head injuries compared to waterskiing injuries [12]. Wakeboarders also suffered more traumatic brain injuries, while waterskiers were more likely to have sprains and strains [12]. Our results are similar to Hostetler et al., and suggest that the majority of injuries associated with waterskiing are sprains and strains; however, our results also demonstrate that lacerations to the face and upper extremity may be on the rise. Therefore, we recommend incorporating facemasks in conjunction with protective headgear to protect waterskiers from face lacerations.

This study has several limitations. Firstly, the NEISS database only includes injuries that resulted in emergency room visits, potentially underrepresenting less severe injuries treated in other settings or those not treated at all. Additionally, the database lacks specific contextual information about the injuries, such as the exact circumstances leading to them or whether the individuals were using protective equipment at the time of the incident. This omission limits the ability to identify specific risk factors and protective measures that could mitigate these injuries. The study's reliance on retrospective data may have led to reporting biases and inaccuracies. Lastly, while the study suggests recommendations for protective gear, it does not provide empirical evidence for the efficacy of such equipment in reducing injury rates, indicating a need for further prospective research to validate these recommendations.

Our study provides an updated comprehensive analysis of waterskiing injuries collected over 10 years. It identifies common injury patterns to the head, face, and knee. Soft tissue injuries, such as strains and sprains, are the most common diagnoses, but head, face, skeletal injuries, and lacerations are also relatively common. Our data in conjunction with that of previous investigations suggest the importance of using protective clothing and devices (e.g., knee guards) and emphasize the need to develop and use ergonomically designed headgear and soft, protective face masks.

## Conclusions

Waterskiing accidents result in a diverse range of injuries that are highly likely to involve the head (i.e., face, brain) in addition to causing strains and sprains. There is a need to investigate prevention strategies and safety measures aimed at reducing the risk, and to develop properly designed and sport-appropriate protective equipment, especially headgear such as face masks, to reduce the rate of waterskiing injuries.
